# 4-Meth­oxy­anilinium iodide

**DOI:** 10.1107/S1600536810047549

**Published:** 2010-11-27

**Authors:** Rui-jun Xu

**Affiliations:** a Ordered Matter Science Research Center, College of Chemistry and Chemical, Engineering, Southeast UniVersity, Nanjing 210096, People’s Republic of China

## Abstract

The crystal structure of the title compound, C_7_H_10_NO^+^·I^−^, displays N—H⋯I hydrogen bonds between the 4-meth­oxy­anilinium cations and the iodide anion together with weaker C—H⋯π contacts.

## Related literature

The title compound was invesitgated as a potential candidate for having good dielectric properties. For compounds with dielectric–ferroelectric properties, see: Hang *et al.* (2009 ▶); Li *et al.* (2008 ▶). 
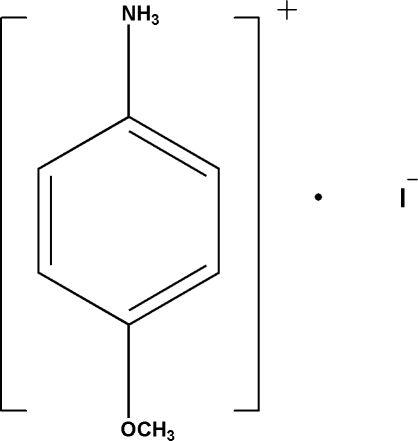


## Experimental

### 

#### Crystal data

C_7_H_10_NO^+^·I^−^
*M* *_r_* = 251.06
Orthorhombic, 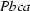

*a* = 12.290 (3) Å
*b* = 7.1302 (14) Å
*c* = 20.304 (4) Å
*V* = 1779.2 (6) Å^3^
*Z* = 8
Mo *K*α radiation
μ = 3.54 mm^−1^
*T* = 298 K
0.40 × 0.30 × 0.20 mm

#### Data collection

Rigaku SCXmini diffractometer
Absorption correction: multi-scan (*CrystalClear*; Rigaku, 2005 ▶) *T*_min_ = 0.291, *T*_max_ = 0.493
16921 measured reflections
2040 independent reflections
1803 reflections with *I* > 2σ(*I*)
*R*_int_ = 0.052

#### Refinement

*R*[*F* ^2^ > 2σ(*F* ^2^)] = 0.032
*wR*(*F* ^2^) = 0.065
*S* = 1.25
2040 reflections
104 parameters
3 restraints
H atoms treated by a mixture of independent and constrained refinement
Δρ_max_ = 0.51 e Å^−3^
Δρ_min_ = −0.45 e Å^−3^

### 

Data collection: *CrystalClear* (Rigaku, 2005 ▶); cell refinement: *CrystalClear*; data reduction: *CrystalClear*; program(s) used to solve structure: *SHELXS97* (Sheldrick, 2008 ▶); program(s) used to refine structure: *SHELXL97* (Sheldrick, 2008 ▶); molecular graphics: *SHELXTL* (Sheldrick, 2008 ▶); software used to prepare material for publication: *SHELXL97*.

## Supplementary Material

Crystal structure: contains datablocks I, global. DOI: 10.1107/S1600536810047549/bg2356sup1.cif

Structure factors: contains datablocks I. DOI: 10.1107/S1600536810047549/bg2356Isup2.hkl

Additional supplementary materials:  crystallographic information; 3D view; checkCIF report

## Figures and Tables

**Table 1:** Hydrogen-bond geometry (Å, °) *Cg*1 is the centroid of the C2–C7 ring.

*D*—H⋯*A*	*D*—H	H⋯*A*	*D*⋯*A*	*D*—H⋯*A*
N1—H1*D*⋯I1^i^	0.86 (3)	2.67 (2)	3.503 (3)	165 (5)
N1—H1*F*⋯I1^ii^	0.86 (1)	2.75 (2)	3.566 (3)	159 (3)
N1—H1*E*⋯I1	0.86 (4)	2.75 (4)	3.568 (3)	159 (4)
C4—H4⋯*Cg*1^iii^	0.93	2.87	3.627 (4)	140
C7—H7⋯*Cg*1^iv^	0.93	2.62	3.483 (4)	155

Symmetry codes: (i) 
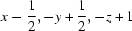
; (ii) 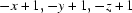; (iii) 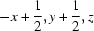; (iv) 
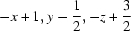
.

**Fig. 1.**
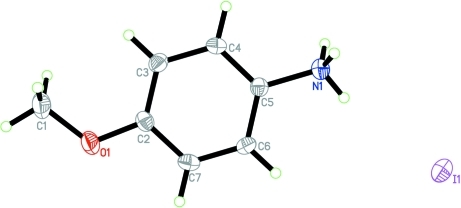
The molecular structure of the title compound, showing the atomic numbering scheme. Displacement ellipsoids are drawn at the 30% probability level.

**Fig. 2.**
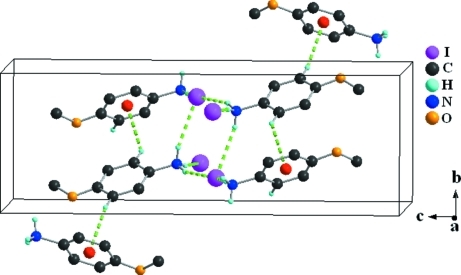
A view of the packing of the title compound, stacking along the b axis. Dashed lines indicate hydrogen bonds.

## supplementary crystallographic information

### Comment

Dielectric-ferroelectric constitute an interesting class of materials, comprising organic ligands (Li *et al.*, 2008), metal-organic coordination compounds (Hang *et al.*, 2009) and organic-inorganic hybrids. We were interested in the title compound as a potential candidate for having good dielectric properties. Unfortunately, the capacitance and dielectric loss measurements did not show any distinct anomaly when observed from 93 K to 455 K (its sublimation temperaure). Regarding its crystal structure, the asymmetric unit of the title compound contains a (4-methoxyanilinium) cation and a iodide anion (Fig.1). The structure is stabilized by C—H···π interactions (C4—H4···*Cg*1 3.627 (4) Å and C7—H7···*Cg*1 3.483 (4) Å) as well as weak N—H···I hydrogen bonds involving the NH_3_ group and linking the cations and anions into a 3D structure (Fig. 2 and Tab. 1).

### Experimental

The title compound was obtained by the addition of hydriodic acid (4.12 ml, 0.022 mol) to a solution of 4-methoxyanilin (2.26 g, 0.02 mol) in ethanol, in the stoichiometric ratio 1.1:1. Good quality single crystals were obtained by slow evaporation after two weeks.

### Refinement

Positional parameters of all the H atoms were calculated geometrically and the H atoms were set to ride on the C and N atoms to which they are bonded, with *U*_iso_(H) = 1.2Ueq(C or N).

### Crystal data

**Table:**

C_7_H_10_NO^+^·I^−^	*F*(000) = 960
*M**_r_* = 251.06	*D*_x_ = 1.875 Mg m^−^^3^
Orthorhombic, *P**b**c**a*	Mo *K*α radiation, λ = 0.71073 Å
Hall symbol: -P 2ac 2ab	Cell parameters from 7161 reflections
*a* = 12.290 (3) Å	θ = 3.0–27.7°
*b* = 7.1302 (14) Å	µ = 3.54 mm^−^^1^
*c* = 20.304 (4) Å	*T* = 298 K
*V* = 1779.2 (6) Å^3^	Prism, colourless
*Z* = 8	0.40 × 0.30 × 0.20 mm

### Data collection

**Table:**

Rigaku SCXmini diffractometer	2040 independent reflections
Radiation source: fine-focus sealed tube	1803 reflections with *I* > 2σ(*I*)
graphite	*R*_int_ = 0.052
Detector resolution: 13.6612 pixels mm^-1^	θ_max_ = 27.5°, θ_min_ = 3.3°
ω scans	*h* = −15→15
Absorption correction: multi-scan (*CrystalClear*; Rigaku, 2005)	*k* = −9→9
*T*_min_ = 0.291, *T*_max_ = 0.493	*l* = −26→26
16921 measured reflections	

### Refinement

**Table:**

Refinement on *F*^2^	Secondary atom site location: difference Fourier map
Least-squares matrix: full	Hydrogen site location: inferred from neighbouring sites
*R*[*F*^2^ > 2σ(*F*^2^)] = 0.032	H atoms treated by a mixture of independent and constrained refinement
*wR*(*F*^2^) = 0.065	*w* = 1/[σ^2^(*F*_o_^2^) + (0.0154*P*)^2^ + 1.2835*P*] where *P* = (*F*_o_^2^ + 2*F*_c_^2^)/3
*S* = 1.25	(Δ/σ)_max_ < 0.001
2040 reflections	Δρ_max_ = 0.51 e Å^−^^3^
104 parameters	Δρ_min_ = −0.44 e Å^−^^3^
3 restraints	Extinction correction: *SHELXL97* (Sheldrick, 2008), Fc^*^=kFc[1+0.001xFc^2^λ^3^/sin(2θ)]^-1/4^
Primary atom site location: structure-invariant direct methods	Extinction coefficient: 0.0053 (2)

### Special details

**Table:**

Geometry. All e.s.d.'s (except the e.s.d. in the dihedral angle between two l.s. planes) are estimated using the full covariance matrix. The cell e.s.d.'s are taken into account individually in the estimation of e.s.d.'s in distances, angles and torsion angles; correlations between e.s.d.'s in cell parameters are only used when they are defined by crystal symmetry. An approximate (isotropic) treatment of cell e.s.d.'s is used for estimating e.s.d.'s involving l.s. planes.
Refinement. Refinement of *F*^2^ against ALL reflections. The weighted *R*-factor *wR* and goodness of fit *S* are based on *F*^2^, conventional *R*-factors *R* are based on *F*, with *F* set to zero for negative *F*^2^. The threshold expression of *F*^2^ > σ(*F*^2^) is used only for calculating *R*-factors(gt) *etc*. and is not relevant to the choice of reflections for refinement. *R*-factors based on *F*^2^ are statistically about twice as large as those based on *F*, and *R*- factors based on ALL data will be even larger.

### Fractional atomic coordinates and isotropic or equivalent isotropic displacement parameters (Å^2^)

**Table:**

	*x*	*y*	*z*	*U*_iso_*/*U*_eq_	
I1	0.613461 (19)	0.22260 (3)	0.471872 (12)	0.04311 (12)	
C1	0.3182 (4)	0.1321 (6)	0.88673 (18)	0.0580 (11)	
H1A	0.3321	0.0724	0.9283	0.087*	
H1B	0.2444	0.1083	0.8736	0.087*	
H1C	0.3293	0.2648	0.8909	0.087*	
C2	0.3836 (3)	0.1268 (4)	0.77653 (15)	0.0332 (7)	
C3	0.3010 (3)	0.2400 (4)	0.75290 (18)	0.0347 (7)	
H3	0.2448	0.2778	0.7806	0.042*	
C4	0.3024 (3)	0.2967 (4)	0.68761 (16)	0.0327 (7)	
H4	0.2472	0.3728	0.6713	0.039*	
C5	0.3856 (2)	0.2399 (4)	0.64723 (16)	0.0303 (7)	
C6	0.4686 (3)	0.1275 (5)	0.67025 (17)	0.0377 (8)	
H6	0.5244	0.0896	0.6423	0.045*	
C7	0.4676 (3)	0.0724 (5)	0.73515 (18)	0.0425 (8)	
H7	0.5237	−0.0019	0.7514	0.051*	
N1	0.3861 (3)	0.2978 (5)	0.57794 (16)	0.0402 (7)	
H1F	0.400 (3)	0.4157 (18)	0.5742 (18)	0.041 (10)*	
H1E	0.438 (3)	0.248 (5)	0.5554 (19)	0.059 (13)*	
H1D	0.324 (2)	0.281 (6)	0.559 (2)	0.077 (16)*	
O1	0.3897 (2)	0.0598 (4)	0.83889 (12)	0.0517 (7)	

### Atomic displacement parameters (Å^2^)

**Table:**

	*U*^11^	*U*^22^	*U*^33^	*U*^12^	*U*^13^	*U*^23^
I1	0.04084 (17)	0.04212 (17)	0.04635 (17)	−0.00309 (9)	0.01153 (9)	−0.00348 (10)
C1	0.078 (3)	0.064 (3)	0.032 (2)	0.008 (2)	0.0061 (19)	0.0060 (18)
C2	0.041 (2)	0.0286 (17)	0.0296 (17)	−0.0015 (14)	−0.0032 (13)	0.0018 (12)
C3	0.0360 (17)	0.0326 (17)	0.0354 (18)	0.0031 (13)	0.0052 (14)	−0.0018 (13)
C4	0.0299 (16)	0.0293 (16)	0.0388 (18)	0.0051 (13)	−0.0023 (13)	0.0015 (13)
C5	0.0321 (17)	0.0284 (16)	0.0303 (16)	−0.0021 (12)	0.0010 (12)	0.0009 (12)
C6	0.0309 (17)	0.042 (2)	0.0404 (19)	0.0093 (14)	0.0065 (14)	0.0018 (15)
C7	0.040 (2)	0.043 (2)	0.044 (2)	0.0164 (16)	−0.0033 (15)	0.0045 (16)
N1	0.0400 (18)	0.0456 (19)	0.0352 (16)	0.0018 (15)	0.0045 (13)	0.0085 (14)
O1	0.0712 (19)	0.0532 (16)	0.0307 (13)	0.0172 (13)	0.0012 (12)	0.0097 (11)

### Geometric parameters (Å, °)

**Table:**

C1—O1	1.407 (4)	C4—H4	0.9300
C1—H1A	0.9600	C5—C6	1.380 (4)
C1—H1B	0.9600	C5—N1	1.466 (4)
C1—H1C	0.9600	C6—C7	1.375 (5)
C2—O1	1.355 (4)	C6—H6	0.9300
C2—C3	1.383 (5)	C7—H7	0.9300
C2—C7	1.386 (5)	N1—H1F	0.860 (10)
C3—C4	1.386 (5)	N1—H1E	0.86 (4)
C3—H3	0.9300	N1—H1D	0.86 (3)
C4—C5	1.371 (4)		
			
O1—C1—H1A	109.5	C4—C5—N1	119.6 (3)
O1—C1—H1B	109.5	C6—C5—N1	119.0 (3)
H1A—C1—H1B	109.5	C7—C6—C5	119.0 (3)
O1—C1—H1C	109.5	C7—C6—H6	120.5
H1A—C1—H1C	109.5	C5—C6—H6	120.5
H1B—C1—H1C	109.5	C6—C7—C2	120.5 (3)
O1—C2—C3	124.8 (3)	C6—C7—H7	119.8
O1—C2—C7	115.2 (3)	C2—C7—H7	119.8
C3—C2—C7	120.0 (3)	C5—N1—H1F	111 (3)
C2—C3—C4	119.5 (3)	C5—N1—H1E	113 (3)
C2—C3—H3	120.3	H1F—N1—H1E	102 (3)
C4—C3—H3	120.3	C5—N1—H1D	113 (4)
C5—C4—C3	119.7 (3)	H1F—N1—H1D	105 (4)
C5—C4—H4	120.1	H1E—N1—H1D	111 (5)
C3—C4—H4	120.1	C2—O1—C1	118.8 (3)
C4—C5—C6	121.3 (3)		
			
O1—C2—C3—C4	−178.6 (3)	N1—C5—C6—C7	−179.6 (3)
C7—C2—C3—C4	0.6 (5)	C5—C6—C7—C2	0.9 (5)
C2—C3—C4—C5	0.0 (5)	O1—C2—C7—C6	178.2 (3)
C3—C4—C5—C6	−0.2 (5)	C3—C2—C7—C6	−1.1 (5)
C3—C4—C5—N1	179.1 (3)	C3—C2—O1—C1	−10.9 (5)
C4—C5—C6—C7	−0.3 (5)	C7—C2—O1—C1	169.8 (4)

### Hydrogen-bond geometry (Å, °)

**Table:**

Cg1 is the centroid of the C2–C7 ring.

**Table:**

*D*—H···*A*	*D*—H	H···*A*	*D*···*A*	*D*—H···*A*
N1—H1D···I1^i^	0.86 (3)	2.67 (2)	3.503 (3)	165 (5)
N1—H1F···I1^ii^	0.86 (1)	2.75 (2)	3.566 (3)	159 (3)
N1—H1E···I1	0.86 (4)	2.75 (4)	3.568 (3)	159 (4)
C4—H4···Cg1^iii^	0.93	2.87	3.627 (4)	140
C7—H7···Cg1^iv^	0.93	2.62	3.483 (4)	155

Symmetry codes: (i) *x*−1/2, −*y*+1/2, −*z*+1; (ii) −*x*+1, −*y*+1, −*z*+1; (iii) −*x*+1/2, *y*+1/2, *z*; (iv) −*x*+1, *y*−1/2, −*z*+3/2.
